# STAT3-mediated Apoptotic-enhancing Function of Sclareol Against Breast Cancer Cells and Cell Sensitization to Cyclophosphamide

**DOI:** 10.22037/ijpr.2020.112587.13843

**Published:** 2020

**Authors:** Havva Afshari, Mitra Nourbakhsh, Niloufar Salehi, Mohammad Mahboubi-Rabbani, Afshin Zarghi, Shokoofe Noori

**Affiliations:** a *Department of Biochemistry, Faculty of Medicine, Shahid Beheshti University of Medical Sciences, Tehran, Iran* *.*; b *Department of Biochemistry, Faculty of Medicine, Iran University of Medical Sciences, Tehran, Iran. *; c *Department of Pharmaceutical Chemistry, School of Pharmacy, Shahid Beheshti University of Medical Sciences, Tehran, Iran.*

**Keywords:** Sclareol, Breast cancer, Apoptosis, STAT3, p53

## Abstract

Sclareol is an organic compound with potential anti-tumor effects against various cancer types. However, its precise molecular mechanism in the suppression of tumor growth has not been fully elucidated. In the present study, the anti-proliferative and apoptosis-inducing effects of sclareol with cyclophosphamide were investigated in breast cancer cells and the involvement of the JAK/STAT pathway was evaluated. For this purpose, MCF-7 breast cancer cells were cultured and treated with various concentrations of sclareol to determine its IC_50_. Cell viability was measured by MTT assay and apoptosis was assessed by flow cytometric analysis of annexin V binding. Gene and protein expression were examined by real-time PCR and Western blotting, respectively. The activity of caspase enzymes was also measured. The results showed that sclareol significantly reduced cell viability and triggered cell death and its co-administration with cyclophosphamide enhanced its anti-cancer properties. Additionally, sclareol up-regulated the expression of p53 and BAX and reduced the expression of Bcl-2. Docking studies indicated an interaction between sclareol and STAT3 which was proved by attenuation of STAT3 phosphorylation after treatment of the cells with sclareol. Sclareol was also capable of suppressing the function of IL-6 in modulating the expression of apoptosis-associated genes. Altogether these data suggest the potential of sclareol as an anti-cancer agent and demonstrate that a combination of sclareol with cyclophosphamide might serve as an effective chemotherapeutic approach resulting in improvements in the treatment of breast cancer.

## Introduction

Breast cancer was the most frequently diagnosed cancer among women worldwide and the leading cause of cancer mortality for women ages 20 and 59 years in the United State in 2018. About 41,000 American female patients were expected to die from breast cancer in 2018 (1, 2). Recent epidemiological data indicate that 1 in 8 women will be diagnosed with breast cancer during her life and its long-term incidence rates are slightly increased by 0.4% per year (3). According to a study, the highest incidence rates of breast cancer were reported in North America and western Europe. However, the global incidence trend of this cancer is rising particularly in countries with a low incidence rate like Iran (4). Treatment options for breast cancer therapy include endocrine therapy for hormone receptor-positive tumors, antibody-based medications, and chemotherapy (5). The same basic categories of systemic therapy are used in metastatic breast cancer; nevertheless, metastatic breast cancer remains incurable in virtually all affected patients and therefore new modalities and medication regimens are needed for the management of breast cancer (5). Recently, there has been a tendency to replace old drug therapy regimens with the newer combination therapy methods (6, 7). In this way, a wide variety of studies have shown that some drugs such as cyclophosphamide can be effective for breast cancer management especially when combined with other anti-cancer agents (8). For example, the docetaxel-cyclophosphamide combination is an efficient and conventionally used chemotherapy strategy in the adjuvant and neoadjuvant regimen for the patients with breast cancer in its early stages (9). Cyclophosphamide is one of the most widely utilized alkylating agents with cytotoxic and immunosuppressive activities typically used in combination with other medications for the treatment of breast cancer (10-12). Moreover, cyclophosphamide was reported to partially exert its anti-tumoral activity by blocking the p-STAT3 pathway (13). 

Sclareol (Labd-14-ene-8, 13-diol) is a labdane diterpene, extracted from *Salvia sclarea*, with anti-cancer properties against various types of cancers (14-16). In breast cancer, sclareol has been shown capable of inhibiting DNA synthesis, inducing cell cycle arrest and triggering apoptosis. Additionally, sclareol has been introduced as a potent enhancer for the activity of well-known chemotherapy agents such as doxorubicin, etoposide, and cisplatin, against breast cancer cells (16). 

Derangement of cytokines is a common finding in breast cancer. Elevation of IL-6 in the tumor microenvironment has been demonstrated in various types of cancers such as breast cancer. IL-6 is able to modify many aspects of tumorigenesis by affecting proliferation, cellular metabolism, survival, apoptosis, angiogenesis, and metastasis through activation of the JAK/STAT3 pathway (17). We had previously shown the effectiveness of sclareol in reducing the tumor volume, suppressing the regulatory T cells and shifting the cytokine profile with decreasing interleukin-4 (IL-4) and augmenting IFN-γ (18). 

In this study, we aimed to investigate the effect of sclareol on cell viability and apoptosis either alone or combined with cyclophosphamide. We also evaluated the effectiveness of sclareol to modulate IL-6-mediated alterations in apoptosis through inhibition of STAT3.

## Experimental

*Molecular docking studies*

To determine whether sclareol is able to directly bind to the SH2 domain of STAT3, the pharmacophore model was built based on the three-dimensional structure of the STAT3β homodimer-DNA complex (PDB: 1BG1) (19). For the generation of the pharmacophore model, the sequence corresponding to residues M586-F716 of STAT3β, comprising its SH2 domain, was taken into account as the receptor. The receptor-based pharmacophore model was created with the software Autodock Vina.

*Cell culture and treatment*

The normal breast cell line, MCF-10A, and the human breast cancer cell line MCF-7, were purchased from Cell Bank of Pasteur Institute (Tehran, Iran). All cell lines were grown in RPMI 1640 medium supplemented with 10% fetal bovine serum (FBS) and penicillin (100 U/ml)/streptomycin (100 μg/mL). All cell lines were then incubated in a humidified incubator with 5% CO2 for 24 h or 48 h at 37 °C. The materials for cell culture were obtained from Gibco, UK. Sclareol, and cyclophosphamide were purchased from Sigma-Aldrich (Germany). For cell treatment, sclareol was dissolved in ethanol and sclareol-free ethanol served as the negative control.

*Cell viability*

Cell viability was determined using the Vybrant MTT cell proliferation assay kit (Thermo Fischer Scientific, USA) containing 3-(4,5-Dimethylthiazol-2-yl)-2,5-diphenyltetrazolium bromide (MTT), as a water-soluble tetrazolium salt, according to the manufacturer’s protocol. Exponentially growing MCF-7 cells were seeded at a density of 2×10^4^ cells/well into 96-well tissue culture plates with a total volume of 200 µl per well. Different concentrations of sclareol (10-150 µM) and cyclophosphamide (5-40 μM) were added. For evaluating the combination of sclareol and cyclophosphamide on cell viability, 8 μM concentration of cyclophosphamide together with 10-40 μM concentrations of sclareol were employed. Thereafter, the cells were incubated for 24 and 48 h at 37°C. The quantity of the formed soluble formazan was calculated through the measurement of absorbance at 490 nm using a plate reading spectrophotometer (PerkinElmer, USA).

*Real-time PCR*

Full-length sequences of the genes of interest including STAT3, JAK2, B cell lymphoma 2 (Bcl-2), Bcl-2 associated X protein (BAX), and p53 were retrieved from NCBI database and recruited for primer design by Primer Express software v1.5 (Applied Biosystems). The primer sequences are presented in Table 1. Total RNA was extracted from the cells using the RNeasy mini kit (Qiagen, Germany), according to the manufacturer’s protocol. M-MLV reverse transcriptase, encoded by Moloney murine leukemia virus (M-MLV RT, Gibco) and oligo-d(T)15 primer (Roche Applied Sciences, Germany) were used to synthesize complementary DNA (cDNA) strand from the 1 µg/ml solution of single-stranded RNA. Real-time PCR analyses were performed on cDNA samples by SYBR-green master mix (Ampliqon, Denmark) using ABI PRISM 7900HT (Applied Biosystems, USA) under the thermocycling conditions as follows: an individual heating phase at 95 °C for 15 minutes, followed by 40 cycles of 95 °C for 15 seconds (denaturation phase) and 60 °C for 30 seconds (annealing phase/extension phase). Melt curve analysis was also performed for each gene to verify the specificity of primers and the lack of non-specific products. The final results were analyzed by the 2^-ΔΔCt^ method with GAPDH as the normalizer. 

*Flow Cytometry*

The effect of sclareol on apoptosis was investigated using a FITC/annexin-V-propidium iodide (PI) kit (apoptosis detection kit; R&D Systems) following the manufacturer’s protocol. MCF-7 cells were treated with sclareol (30 µM), cyclophosphamide (8 μM) or their combination. Subsequently, the treated cells were centrifuged (1000 g) for 5 min at room temperature (18-24 °C), washed once with 5 mL phosphate-buffered saline (PBS) and then resuspended in binding buffer. Five microliters of FITC-annexin V and 5 µL of PI were added to the cell suspension and incubated for 10 min in the dark at room temperature. Analysis of FITC-Annexin V binding was carried out on a FAC scan flow cytometer (BD Biosciences) with an emission wavelength of 350 nm and an excitation wavelength of 488 nm. The tests were performed in three independent experiments. Annexin V positive /PI negative cells were reported as early apoptotic cells while those positively stained by both Annexin V and PI were considered as late apoptotic cells. Annexin V negative/ PI-positive cells were presented as necrotic cells. 

*Western blot analysis*

MCF-7 cells were seeded in six-well culture plates and allowed to adhere to the plate overnight. Subsequently, they were then incubated with the sclareol solution (30 μM). A group of cells was additionally treated with IL-6 (50 ng/mL) 4 hours after treatment with sclareol. The cells were harvested by centrifugation at 412 g for 10 min, washed twice with PBS and suspended in RIPA lysis buffer (Thomas Scientific Inc., USA) for preparation of whole-cell lysates. The lysates were centrifuged and the supernatant was used for Western blot analysis. Bicinchoninic acid (BCA) assay kit (Thermo Fisher Scientific, UK) was used to measure the protein content. The samples were subsequently loaded into 10 % SDS-PAGE (40 µg total protein/lane) and transferred onto a polyvinylidene fluoride (PVDF) membrane (Millipore, USA) by electroblotting. The membranes were blocked with 5% non-fat milk at room temperature for 1 h. The membranes were then incubated overnight at 4°C with 1:1000 dilution of primary antibodies against STAT3 (Cell Signaling, Danvers, USA), phosphorylated STAT3 (Cell Signaling, Danvers, USA), and β-actin (Sigma-Aldrich, Germany). The membranes were incubated with corresponding horseradish peroxidase (HRP)-conjugated anti-mouse IgG (Santa Cruz Biotechnology, UK) for 1 h at room temperature, in the dark. Immunoblots were detected using an enhanced chemiluminescent kit (SuperSignal, Thermo Fisher Scientific, UK). The density of the visualized bands was quantitated using ImageJ software (NIH, Bethesda, USA).

*Measurement of caspase activity*

A colorimetric assay was used to determine caspase activity. For this purpose, MCF-7 cells were lysed in lysis buffer (250 mM sucrose, 0.02 M Tris HCl pH 7.4, 1% Triton X-100, 150 mM NaCl, 1 mM EDTA, 1 mM EGTA, and 1 mM DTT) at 4 °C for 30 min while vortexing. A 200-μg sample of cell lysate protein was mixed in assay buffer (25 mM HEPES pH 7.5, 0.1% CHAPS, 5% sucrose, 5 mM DTT, and 2 mM EDTA) in a final volume of 100 μl, followed by addition of 10 μl of 2 mM of the substrate caspase-8 (Z-IETD-pNA), caspase-9 (Ac-LEHD-pNA), or caspase-3 (Z-DEVDpNA) for the respective caspase assay. The reaction mixture was incubated at 37 °C for 30 min and liberated p-nitroaniline (pNA) was measured at 405 nm with a SpectraMAX 190 Microplate Reader (Sunnyvale, CA, USA).

*Statistical Methods*

Data are expressed as the mean ± standard error of the mean. Statistical data analyses were carried out using one-way analysis of variance (ANOVA) followed by Duncan’s multiple range test for post-hoc evaluation. SPSS 17.0 software was used for the analysis of acquired data. For all analyses, *P*<0.05 was considered to indicate a statistically significant difference.

## Results

*Sclareol reduced cell viability and potentiated the cytotoxic activity of cyclophosphamide on breast cancer cells*

**Figure 1:**
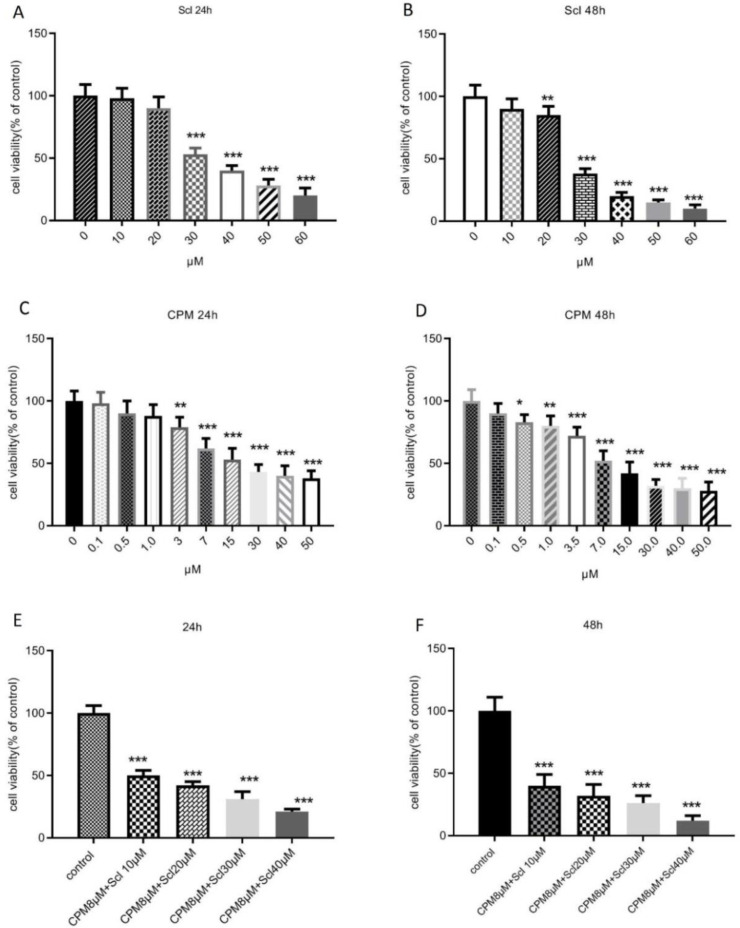
The effect of sclareol, after 24 h (A) and 48 h (B), cyclophosphamide after 24 h (C) and 48 h (D), or their combination after 24 h (E) and 48 h (F), on the viability of MCF-7 cells. Scl: sclareol; CPM: cyclophosphamide. The data shown are the mean ± SD of at least three separate experiments. The values were compared with the control. Treatment with IL-6 served as positive control

The cytotoxic effect of sclareol on breast cancer cells was determined based on the levels of formazan formation in MTT assay. As shown in Figures 1A and B, sclareol caused a dose-dependent reduction of the survival of MCF-7 cells after 24 hours which became significant at the doses higher than 30 μM. After 24 h of treatment, the IC_50_ value of sclareol was 31.11 μM and further decreased to 27.65 μM after 48 h. The effect of cyclophosphamide on cell viability was also evaluated and it was shown that 24 h treatment of MCF-7 cells with cyclophosphamide caused a significant decline in the survival of MCF-7 breast cancer cells at the dose of 3 μM with an IC_50_ value of 8.3 μM (Figure 1C). When the incubation time with cyclophosphamide was extended for 48 h, the viability was further decreased and the IC_50_ value diminished to 7.6 μM (Figure 1D). The lowest effective dose of cyclophosphamide in decreasing the viability was 0.5 μM after 48 hours of treatment.

In the next step cell viability was assessed in response to the combination of cyclophosphamide and sclareol. As it is presented in Figure 1E, co-administration of sclareol and cyclophosphamide resulted in a more remarkable reduction in cell survival compared to cyclophosphamide alone in MCF-7 cells. Increasing the dose of sclareol combined with 8 μM cyclophosphamide further diminished cell viability (Figure 1F). 

*Induction of apoptosis by sclareol alone or combined with cyclophosphamide*

Apoptosis is the major hallmark of cancer cell response to anticancer therapies. Hence, apoptosis was analyzed in cells treated with sclareol, cyclophosphamide, or the combination of both compounds. 

**Figure 2:**
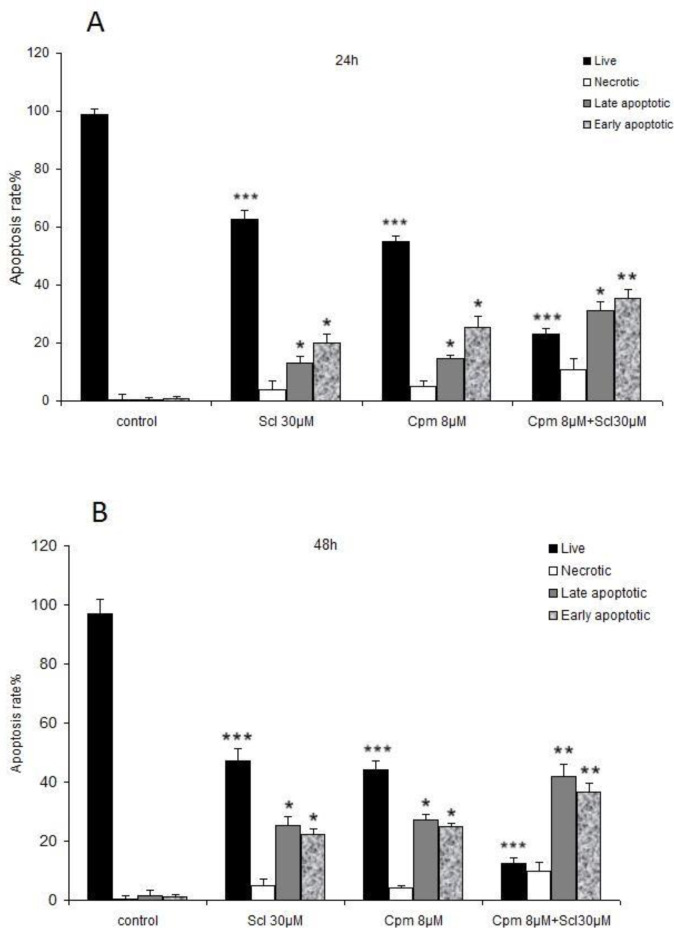
The results of the flow cytometric analysis of the apoptosis in MCF-7 cells treated with sclareol (Scl) (30 µM), cyclophosphamide (Cpm) (8 μM) and their combination, compared with control cells

As it is shown in Figures 2 A and B, a significant increase in both the early and late-stage apoptotic cells was observed after incubation with 30 μM of sclareol both for 24 and 48 hours. Co-administration of sclareol and cyclophosphamide increased the population of cells in both early and late apoptosis compared to the control cells (Figures 2 A and B).

*Induction of apoptotic genes in response to sclareol*

Sclareol was found to promote apoptosis of the breast cancer cells; thus, the expression of various apoptosis-related genes such as p53, Bcl-2, and Bax, as well as JAK2 and STAT3 was analyzed to achieve a better understanding of how sclareol exerts cytotoxic effects and promotes apoptosis. 

**Figure 3:**
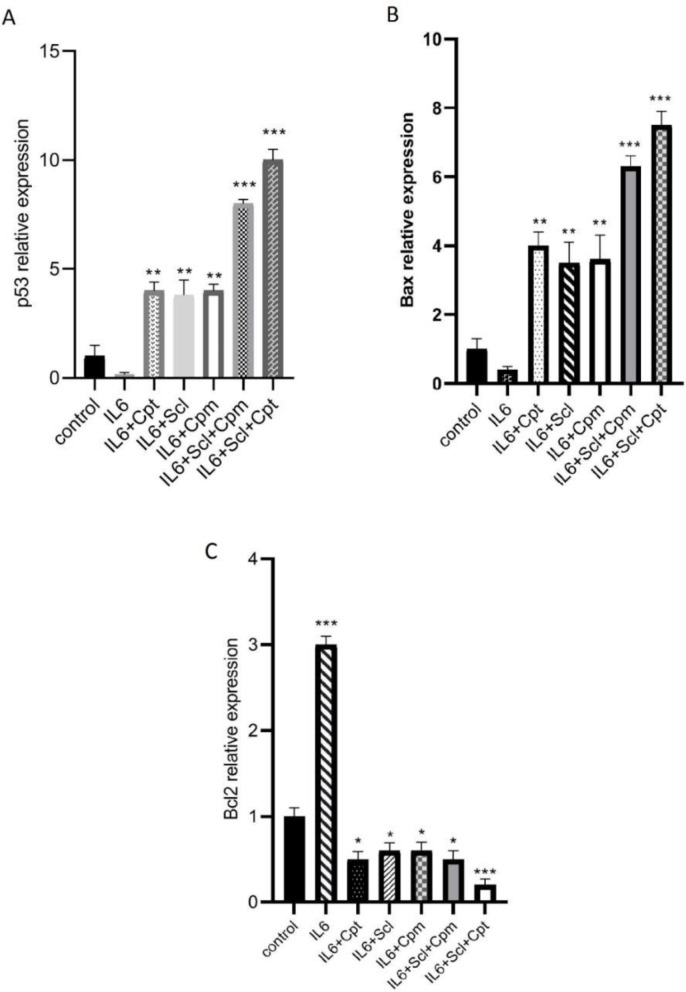
The effect of sclareol (Scl) alone or in combination with either cyclophosphamide (Cpm) or cryptotanshinone (Cpt) on IL-6-induced modulation of apoptotic genes expression including p53 (A), BAX (B) and Bcl-2 (C). The presented data are mean ± SD of at least three separate experiments. The obtained values were compared with the control. Treatment with IL-6 served as positive control

First of all MCF-7 breast cancer cells were treated with IL-6 which is an established promoter of breast cancer progression and its inhibition leads to the induction of apoptosis (20). Furthermore, the expression levels of IL-6 were found to be significantly lower in MCF-7 than some other cancer cell lines like MDA-MB-231 (21). As it is shown in Figure 3A, p53 expression level was significantly diminished after the treatment of the cells with IL-6. When sclareol was added to the cells it could significantly abrogate the suppressive effect of IL-6 on p53 and significantly enhanced p53 expression. Similar outcomes were also reported from cyclophosphamide. The combination of sclareol and cyclophosphamide was even more effective in the induction of p53 expression and repression of the effect of IL-6 (Figure 3A). 

On the other hand, since JAK/STAT pathway is an efficient pathway to suppress cancer cell growth and IL-6 exerts its effect on breast cancer cells via this pathway, a group of cells was treated by a combination of IL-6, sclareol, and cryptotanshinone, as the STAT3 inhibitor, and the highest increase in p53 expression was observed in this group indicative of the involvement of JAK/STAT pathway in the function of sclareol. 

IL-6 was also able to inhibit the expression of BAX, an efficient proapoptotic factor, while it induced the expression of Bcl-2 as a potent anti-apoptosis factor (Figure 3 B, C). Sclareol inhibited the effect of IL-6 and was able to significantly induce Bax expression (Figure 3 B). 

The expression of Bcl2 which was increased by IL-6 was significantly suppressed when sclareol was added to the cells (Figure 3C). Cyclophosphamide also changed the effect of IL-6 both on BAX and Bcl-2. When cyclophosphamide was combined with sclareol, it exerted a significant provocative effect and significantly increased BAX expression (Figure 3B). Inclusion of cryptotanshinone in the cellular treatments, enhanced the effect of sclareol on IL-6-induced modulation of both Bcl-2 and BAX, pointing out the role of JAK/STAT pathway in this outcome (Figure 3 B, C). 

*Enhancement of the JAK/STAT pathway by sclareol*

As mentioned above, sclareol could inhibit the effects of IL-6 on gene expression. Since IL-6 functions through the JAK/STAT pathway, it was hypothesized that sclareol could modify the STAT3 expression and activity. The mRNA expression of both STAT3 and JAK2 was analyzed after treatment with sclareol, cyclophosphamide or cryptotanshinone together with IL-6. The expression of neither JAK2 nor STAT3 was affected by IL-6. The addition of sclareol either alone or combined with cyclophosphamide or cryptotanshinone could not change the gene expression of these two signaling proteins showing that their regulation by IL-6 and sclareol are not achieved at the transcriptional level.

**Figure 4:**
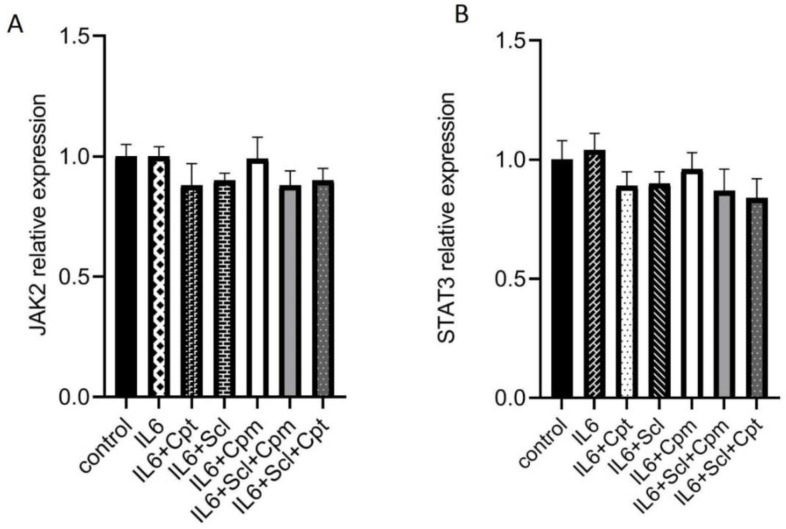
The effect of sclareol (Scl) in combination with IL-6, cryptotanshinone (Cpt) and cyclophosphamide (Cyc) on the gene expression of JAK2 (A) and STAT3 (B) compared to control group and the cells treated with IL-6. The presented data are the mean ± SD of at least three separate experiments

Neither combination was effective on the gene expression of STAT3 and JAK2 (Figure 4A, B), indicating that none of these compounds modulate STAT3 and JAK2 at the transcriptional level. 

**Figure 5:**
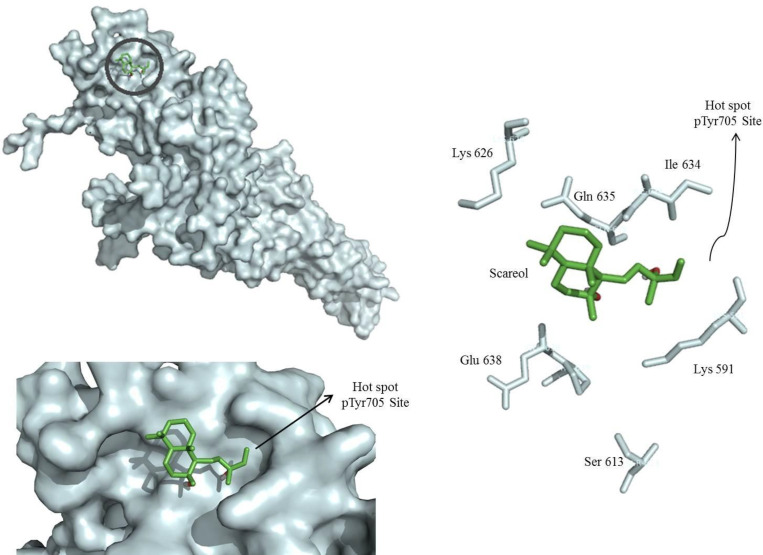
Molecular docking model of sclareol (Scl) binding to the STAT3 SH2 domain (PDB: 1BG1) generated by autodock vina. The surface of the SH2 domain was colored pale cyan and the key residues are shown. Sclareol bound to hot spot pTyr705 site of STAT3 SH2 domain

Taking into account the possibility of post-translational regulation of STAT3 by sclareol, docking studies were performed to evaluate the interaction between sclareol and STAT3. As shown in Figure 5, sclareol was perfectly docked into the native hot spot pTyr705 site and side pocket of the SH2 domain of STAT3 with a predicted binding energy of 5.4 kcal/mol which stabilized the enzyme-inhibitor complex. Therefore, sclareol was speculated to be a direct inhibitor of STAT3 by interacting with the SH2 domain of this protein. 

STAT3 is activated by phosphorylation (22). Hence, in the next step, MCF-7 cells were treated with sclareol and the phosphorylation of STAT3 was evaluated by Western blotting using specific antibody against the phosphorylation form of STAT3 and compared with other treatments including cyclophosphamide as well as IL-6 and cryptotanshinone as the inducer and inhibitor of STAT3, respectively. 

**Figure 6.**
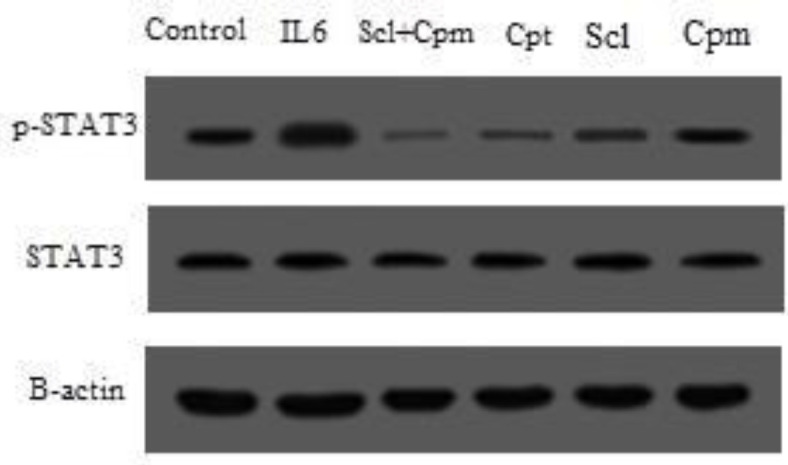
The phosphorylation status of STAT3 after treatment of cells with sclareol (Scl) either alone or combined with cyclophosphamide (Cyc) or cryptotanshinone (Cpt). Untreated cells that received only the solvent (ethanol) were used as negative control and treatment with IL-6 served as the positive control

As shown in Figure 6, treatment with IL-6 caused a remarkable increase in the phosphorylated form of STAT3 while cryptotanshinone suppressed the phosphorylation of STAT3. Sclareol was also able to significantly decrease STAT3 phosphorylation, an effect that was augmented by the addition of cyclophosphamide so that co-administration of sclareol and cyclophosphamide was even more effective in suppression of STAT3 phosphorylation compared to each separate treatment (Figure 6). 

*Activation of caspases 8 and 9 by sclareol*

**Figure 7:**
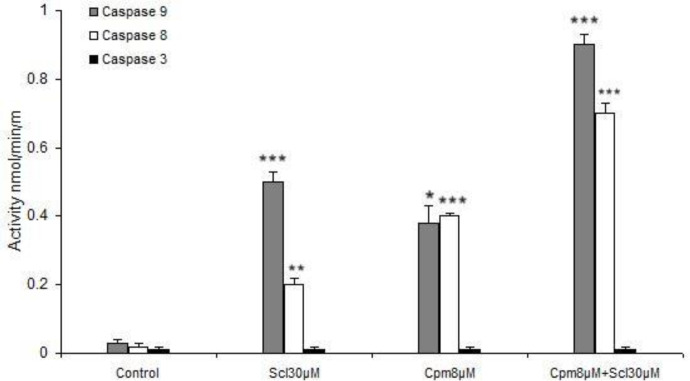
Activation of caspases 8 and 9 but not 3 by sclareol (Scl) and cyclophosphamide (Cyc). The presented data are the mean ± SD of at least three separate experiments. * *P*<0.05, ** *P*<0.01, *** *P*<0.001

As mentioned above, sclareol was able to efficiently induce apoptosis and enhance the expression of apoptosis-associated factors. Since the main apoptotic pathways are executed by caspases including 8, 9 and 3, the activities of these caspases were evaluated in response to sclareol. Both cyclophosphamide and sclareol effectively activated caspases 8 and 9 (Figure 7). Sclareol activated caspase 9 more efficiently compared to caspase 8 while cyclophosphamide was equally effective on both caspases 8 and 9. Cyclophosphamide and sclareol had an additive effect on the activation of caspases. Since caspase 3 is not generally expressed in MCF-7 cells, its activity remained unchanged in all treatments (Figure 7). 

## Discussion

Human breast cancer is a complex illness caused by the joint interaction of genetic, epigenetic, and environmental factors (23). Despite considerable developments in the treatment of breast cancer, current conventional therapies for this disease still have major limitations (24). Thus, novel therapeutic strategies are needed to effectively modulate various carcinogenic mechanisms. Natural compounds such as diterpenes, play an important role in chemoprevention and in improving the effectiveness of cancer treatment by modifying the sensitivity of cancer cells to chemotherapeutics (25). 

Sclareol, as a diterpene, is a renowned phytochemical that was found to be able to potently reduce cell viability and induce apoptosis with an IC_50 _at the micromolar level in estrogen receptor (ER)-positive MCF-7 cells (26). 

Compelling experimental evidence from both clinical trials and animal studies demonstrated that the JAK2/STAT3 pathway plays a pivotal role in the initiation, progression, and metastasis of breast cancer (27, 28). The present study aimed to find out the molecular mechanism underlying sclareol anticancer activity, particularly in regards to its effects on the JAK2/STAT3 signaling pathway. In fact, on the basis of the existing evidence, we hypothesized that bioavailable sclareol alone, or in combination with cyclophosphamide may modulate breast carcinogenesis, primarily by inhibiting Stat3 activation. 

*Sclareol effects on cell viability and apoptosis*

In this study, we showed that sclareol could effectively diminish cell viability and induce apoptosis in breast cancer cells at a concentration of 30 μM. In line with our findings, antiproliferative and apoptosis-inducing effects against breast cancer cells have been previously described for sclareol (29). It has also been shown to reduce cell survival and induce cell death in other cancer types such as cervical cancer and osteosarcoma (30, 31). Sclareol has been reported to sensitize cancer cells to various anticancer drugs. For example, sclareol enhances the efficacy of doxorubicin, etoposide, and cisplatin, against breast cancer cells (29) and bortezomib in cervical cancer (31). In the current study, we showed for the first time that co-administration of sclareol and cyclophosphamide exhibited a much more potent anti-proliferative effect against breast cancer cells and induced apoptosis more competently, suggesting that the combination of sclareol and cyclophosphamide might be considered as a suitable candidate for breast cancer treatment.

Apoptosis, the major type of programmed cell death, is an active cell suicide process. One of the major hallmarks of carcinogenesis is a circumvention of apoptosis by cancer cells. On the other hand, cancer cell death is an essential part of different strategies that exist for the elimination of malignant cells. Thus, initiation of cell death pathways as well as targeting the lesions that suppress cell death are exciting remedies for cancer treatment (32). 

**Figure 8:**
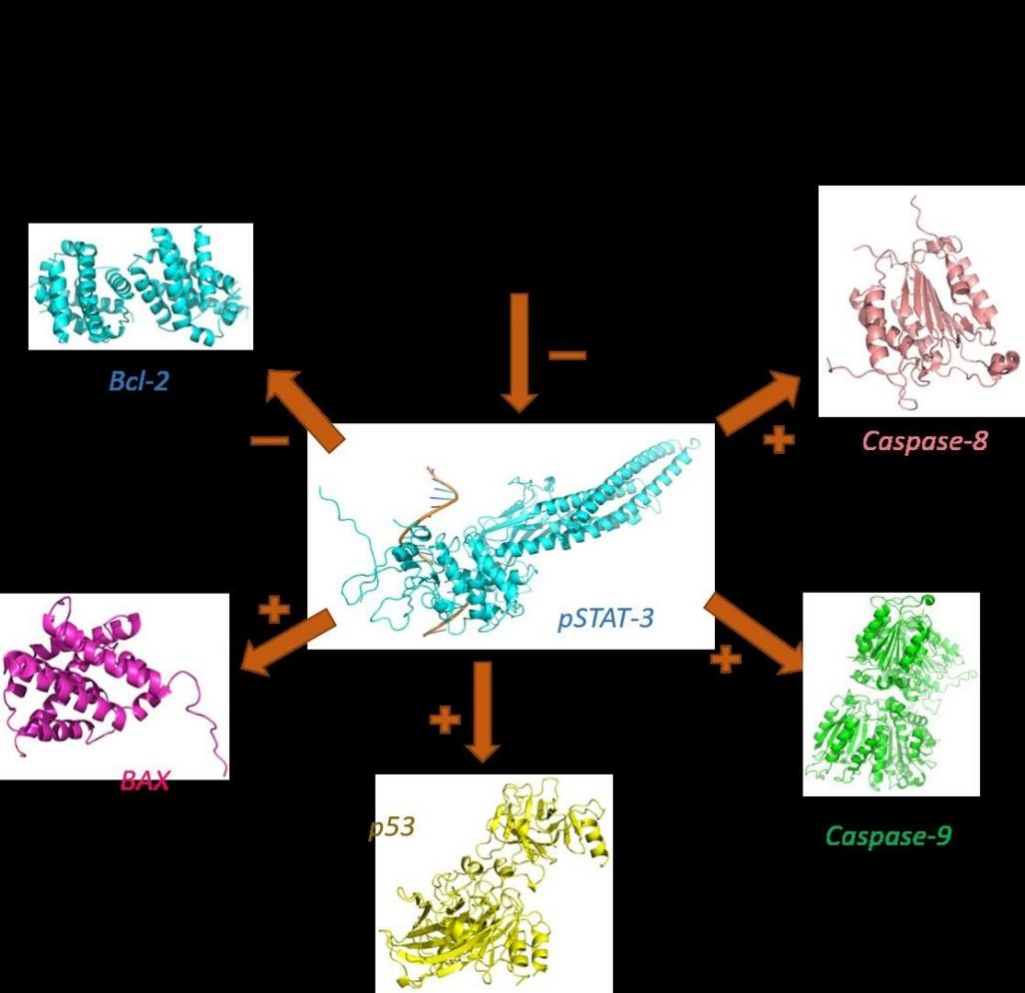
The pro-apoptotic and anti-apoptotic factors modulated by sclareol in MCF-7 cells

In this study, we showed for the first time that sclareol significantly enhanced the expression of major pro-apoptotic proteins including p53 and BAX and attenuated the expression of Bcl-2 as the chief anti-apoptotic factor (Figure 8). 

In addition to serving as the promoter of metastasis and a regulator of cell invasion (33, 34), Bcl-2 is known to be involved in the mediation of chemotherapy resistance in some types of cancers (35-38). Accordingly, sclareol may have the potential to overcome cancer chemoresistance by inhibiting Bcl-2. Consistently, the up-regulation of BAX and suppression of Bcl-2 by sclareol has been previously reported in osteosarcoma cells (14). Nevertheless, sclareol has exhibited apoptosis-promoting properties in p53-null cell lines (29) and so it seems that other pathways might also be involved in the efficacy of sclareol in the induction of cell death. Anyhow, further investigation is required to clarify the detailed mechanism of these effects by sclareol.

Here we revealed that caspase 8 was activated by sclareol which suggests the stimulation of the extrinsic pathway of apoptosis by this compound. It has been established that in case of high levels of activated caspase 8, the effector caspases 3, 6, and 7 are triggered, while in some other cell types, activation of caspase 8 leads to cytochrome c release from mitochondria which forms the apoptosome complex that results in stimulation of caspase-9 (39). Caspase-9 has been described as the initiator of the intrinsic pathway that binds to Apaf-1 and cytochrome c and then activates caspase-3 and subsequently leads to apoptosis (40). We found out that caspases 8 and 9 were activated by sclareol. Thus, based on our findings it seems that activation of caspases in breast cancer cells in response to sclareol follows the latter mechanism and happens through the cross-talk between extrinsic and intrinsic pathways of apoptosis. Caspase 3 is not normally expressed in MCF-7 breast cancer cells (41), hence its expression was not induced in different experimental conditions. 

Meanwhile, the activities of caspase-8 and -9 were also markedly increased in the MCF-7 cell line co-treated with these two compounds comparing to that of either agent alone (Figure 7). In addition, we observed that the co-treatment more greatly decreased the expression of Bcl-2 and increased expressions of Bax, compared to either agent treated alone. 

*Sclareol effects on the JAK2/STAT3 path-way*

STAT3 is persistently activated in numerous cancers and is involved in cancer cell proliferation, invasion, and migration. Additionally, it tempers epigenetic modification, induces epithelial-mesenchymal transition and promotes cancer stem cells self-renewal and differentiation (22). 

Therefore, the present study aimed to elucidate the molecular mechanism underlying sclareol anticancer activity, particularly in regards to its effects on the JAK2/STAT3 signaling pathway. We showed that sclareol could interact with STAT3 and attenuate its phosphorylation. As reported in previous studies, high concentrations of phosphorylated STAT3 (p-STAT3) was reported to be associated with high metastatic rate of a majority of human cancers (42-44) such as lung (45), ovarian (46), colorectal cancers (47) and TNBC (48) and their poor survival outcomes. In addition, as described previously, accumulating evidence suggests that STAT3, as a critical multifunctional mediator plays a crucial role in the regulation of angiogenesis and cell adhesion under both physiological and pathological conditions (49-51). However, the expression levels of non-phosphorylated JAK2 and STAT3 themselves were not affected by sclareol, suggesting that sclareol may intervene principally at the post-translational phase. 

Additionally, we found that the modulatory functions of IL-6 on the expression of apoptotic genes were impeded by sclareol. Considering the role of IL-6 on the initiation of the JAK/STAT pathway, inhibition of the IL-6 function by sclareol further verified the effect of this compound on hindering the STAT3 phosphorylation and activity. The addition of cryptotanshinone, a well-characterized inhibitor of STAT3, to the cells treated by sclareol enhanced the inhibitory effect of sclareol and confirmed that the effects of sclareol on breast cancer cells are mediated through JAK/STAT pathway. 

Interestingly it was found that combined treatment of sclareol and cyclophosphamide attenuated STAT-3 phosphorylation compared with groups of sclareol and cyclophosphamide treated alone in MCF-7 cell line. 

Taken together, sclareol may be considered as a new promising lead for the development of active anticancer agents. However, many of the properties of sclareol are still unknown. Hence, further preclinical and clinical studies are needed to figure out sclareol toxicity as well as its pharmacokinetic profiles, before introducing an officially-marketed medicinal product of this compound. 

**Table 1:** Primer sequences

**Primer name**	**Sequence**
JAK2	F: 5'-GGGAGGTGGTCGCTGTAAAA-3' R: 5'-ACCAGCACTGTAGCACACTC-3'
STAT3	F: 5'-GGTTGGACATGATGCACACTAT-3' R: 5'-AGGGCAGACTCAAGTTTATCAG-3'
Bcl-2	F: 5'-GGTGGGGTCATGTGTGTGG-3' R: 5'-CGGTTCAGGTACTCAGTCATCC-3'
BAX	F: 5'-GATGCGTCCACCAAGAAGC-3' R: 5'-AAGTCCAATGTCCAGCCCAT-3'
p53	F: 5'-CAGCACATGACGGAGGTTGT-3' R: 5'- TCATCCAAATACTCCACACGC-3'

## Conclusion

In conclusion, our findings convincingly provided evidence for the efficacy of sclareol in attenuation of cell viability and inducing apoptosis in MCF-7 breast cancer cell line. Furthermore, for the first time, it was found that the addition of sclareol to cyclophosphamide treatment increased the potency of this anti-cancer agent via the induction of apoptotic cell death as well as inhibition of STAT3 phosphorylation. The co-treatment-induced apoptosis was likely attributable to the activation of p53, Bax, caspase 8, and caspase 9 as well as suppression of Bcl-2. The in-vitro results also validated the results of the molecular docking study. Hence, sclareol can be considered as a suitable candidate for therapeutic interventions. Further animal studies and human clinical trials are required to develop sclareol as a treatment option against breast cancer. 
